# Supplementary material for: Expectations versus Reality of Designer Dog Ownership in the United States
Source: Animals (Basel). 2022 Nov 23;12(23):3247. doi: 10.3390/ani12233247 (PMC9736103; doi:10.3390/ani12233247)
Supplement: Supplementary file 1 [file animals-12-03247-s001.zip › animals-1989659-supplementary.pdf]

Supplementary File S1

Survey Questions

Basic question: What is your dog's name? If you have more than one dog, please use the dog that you have had the longest.

Section I: Expectations Versus Reality (from Packer et al., 2019)

1. Are the veterinary costs related to owning your dog…
   a. Less than expected
   b. Met expectations
   c. More than expected
2. Are the exercise levels needed by your dog…
   a. Less than expected
   b. Met expectations
   c. More than expected
3. Are your dog's overall behaviors…
   a. Worse than expected
   b. Met expectations
   c. Better than expected

Part II Perceived Costs (from Monash Dog Owner Relationship Scale, Dwyer et al., 2015).

These questions will be on a 5-point scale ranging from "never" to "very often" and from "completely disagree" to "completely agree".

1. How often do you feel that looking after your dog is a chore?
2. It is annoying that I sometimes have to change my plans because of my dog.
3. It bothers me that my dog stops me doing things I enjoyed doing before I owned it.
4. There are major aspects of owning a dog I don't like.
5. How often does your dog stop you doing things you want to do?
6. My dog makes too much mess.
7. My dog costs too much money.
8. How hard is it to look after your dog?
9. How often do you feel that having a dog is more trouble than it is worth?

Part III: Behavior (from Bouma et al., 2020a)

5-point scale including "never", "sometimes", "regularly", "often", and "very often". Participants will answer these questions for how they expected their dog to be, and then will answer them for how their dog actually acts.

1. Not responding to stop commands
2. Bad eating manners
3. Not listening when called upon
4. Pulling the leash
5. Inappropriate soiling
6. Inappropriate soft/playful biting
7. General disobedience
8. Aggression towards other dogs
9. Aggression towards unfamiliar people
10. Aggression towards familiar people
11. Inappropriate digging
12. Destructive behavior
13. Inappropriate chewing or biting inedible objects
14. Hyperactivity
15. Inappropriate barking
16. Jumping onto people

Part IV: Satisfaction with Type of Dog (from Bouma et al., 2020b)

5-point scale from “completely disagree” to “completely agree”

1. I am satisfied with my decision
2. I am confident I made a deliberate decision
3. It would have been useful if I had asked for more advice before acquiring my dog
4. If I were to acquire another dog in the future, I would prepare myself differently
5. The decision to acquire my dog was the right decision for my current situation
6. The decision to acquire my dog did not play out as expected
7. I sometimes have mixed feelings about the decision to acquire my dog

Part V: Dog Acquisition Decision (Response options from Packer et al., 2017)

Participants will rate how much each of the following factors influenced their decision to acquire their dog. They will rate each factor on how influential they were on their decision on a scale of 0-4, with 0 being not influential at all, and 4 being very influential.

1. Appearance
2. Popularity of the breed
3. Childhood experiences
4. Good dog breed for children
5. Good companion breed
6. Working ability
7. Exercise encouragement

8. Cost
9. Generally healthy breed
10. Long life expectancy
11. Breed suited to lifestyle
12. Breed is easy to take care of
13. Recommended by friend or family member
14. Recommended by a veterinary professional
15. Celebrity endorsement/ownership

Section VI: Basic Information

1. What is your gender?
    a. Male
    b. Female
    c. Non-binary
    d. Prefer not to say
2. What is your age? Choose your exact age from the drop-down menu.
3. What is your race?
    a. Asian or Pacific Islander
    b. Black or African American
    c. Hispanic or Latino
    d. Native American or Alaskan Native
    e. White or Caucasian
    f. Multiracial or Biracial
    g. A race/ethnicity not listed here
4. Do you have children in the home?
    a. Yes
    b. No
5. What type of dog do you have? If you have more than one dog, please use the dog that you have had the longest.
    a. Hybrid dog (the intentional mix of several dog breeds, e.g., goldendoodle, labradoodle, cockapoo, etc.)
    b. Purebred dog
    c. Mixed breed not typically considered a hybrid
6. If you answered hybrid dog, please specify what kind.
7. If you answered hybrid dog, please specify the generation, if known.
8. If you answered purebred dog, please specify the breed.
9. If you answered mixed breed typically not considered a hybrid, please specify mix of breeds or “unknown”.
10. Is this the first dog that you have owned?
    a. Yes
    b. No
11. Have you owned this type of dog before?
    a. Yes

b. No

12. Where did you acquire your dog?
    a. Breeder
    b. Pet store
    c. Shelter or rescue
    d. Friend or family member
    e. Other (please specify)
13. If you answered breeder, did you meet the parents of your dog?
    a. Yes
    b. No
14. If you answered breeder, where did you pick your dog up for the first time?
    a. On-site at breeding facility
    b. Off-site location
15. Did you speak to any professionals about your breed choice before acquisition?
    a. Yes
    b. No
16. If yes, who did you speak to? Check all that apply.
    a. Veterinarian
    b. Professional trainer
    c. Breeder
    d. Other

17. Does your dog meet the wishes that you had when you acquired it? Please list up to 3 ways that your dog has met your expectations and up to 3 ways that they have not met your expectations.
